# Nervousness and Nervines

**Published:** 1877-05

**Authors:** 


### NERVOUSNESS AND NERVINES.

Nervousness is one of the prices we have to pay for civilization; the nervous savage is a being unheard of. For this disorder, which is partly of mental and partly of bodily nature, relief is sought in various ways, and among these we may place the employment of narcotics. The temporary relief afforded by these drugs is very apt to lead those who suffer from nervous sensations to put too much trust in and resort too frequently to them. In the long run they prove most destructive to health. Their use has of late become so frequent as to threaten society with a serious evil. It has been boldly contended that chloral is to be found in the work-boxes and baskets of nearly every lady in the west end of the metropolis, "to calm her nerves." No doubt this is an exaggeration, but it is a fact that New York chloral punch had become an institution scarcely a year after the introduction of chloral into medical practice, and now it turns out that Germany—"sober, orderly, paternally-ruled Germany"—has such a thing as morphia disease spreading among its population. The symptoms are not unlike those of opium eating. Experience suggests that persons suffering from this disease should at once be deprived of the drug. Their willfulness and liability to relapse, however, are so great, that it is said that only about twenty-five per cent. have been seen to recover in a large series of cases.—*Cassell's Magazine.*

